# How to recover from a bad start: size at metamorphosis affects growth and survival in a tropical amphibian

**DOI:** 10.1186/s12898-020-00291-w

**Published:** 2020-04-21

**Authors:** Diana Székely, Dan Cogălniceanu, Paul Székely, Diego Armijos-Ojeda, Valentina Espinosa-Mogrovejo, Mathieu Denoël

**Affiliations:** 1 Departamento de Ciencias Biológicas, EcoSs Lab, Universidad Técnica Particular de Loja, Loja, Ecuador; 2 Faculty of Natural and Agricultural Sciences, Ovidius University Constanța, Constanța, Romania; 3 Laboratory of Ecology and Conservation of Amphibians (LECA), Freshwater and OCeanic Science Unit of ReSearch (FOCUS), University of Liège, Liège, Belgium; 4 Asociation Chelonia, Bucharest, Romania; 5 Programa de Doctorado en Conservación de Recursos Naturales, Universidad Rey Juan Carlos, Móstoles, Spain

**Keywords:** Adaptive plasticity, Growth compensation, Life cycles, Life-history, Metamorphosis

## Abstract

**Background:**

In species with complex life cycles, size at metamorphosis is a key life-history trait which reflects the complex interactions between costs and benefits of life in the aquatic and terrestrial environments. Whereas the effects of a deteriorating larval habitat (e.g. pond desiccation) on triggering an early metamorphosis have been extensively investigated in amphibians, the consequences of the resulting reduced size at metamorphosis on fitness in the post-metamorphic terrestrial stage remain poorly understood. We tested the hypothesis that a smaller size at metamorphosis negatively affects performance and survival in the ensuing terrestrial stage. Using as model a tropical amphibian (*Ceratophrys stolzmanni*) showing a large phenotypic plasticity in metamorphosing traits, we evaluated the effects of size at metamorphosis on fitness-related trophic and locomotor performance traits, as well as on growth and survival rates.

**Results:**

Our results support the hypothesis that a larger size at metamorphosis is correlated with better survival and performance. The survival rate of large metamorphosing individuals was 95%, compared to 60% for those completing metamorphosis at a small size. Locomotor performance and gape size were positively correlated with body size, larger animals being more mobile and capable to ingest larger prey. However, smaller individuals achieved higher growth rates, thus reducing the size gap.

**Conclusions:**

Overall, size at metamorphosis affected profoundly the chances of survival in the short term, but smaller surviving individuals partly compensated their initial disadvantages by increasing growth rates.

## Background

Species with complex life cycles, such as biphasic amphibians and insects, are able to exploit different ecological niches and optimize their life-history in discrete developmental stages [1, 2]. The transition occurring at metamorphosis usually requires dramatic and irreversible morphological transformations, and is frequently accompanied by a complete change of the ecological niche [3, 4]. Pond-breeding amphibians, which in their post-larval stages become terrestrial, represent ideal models to investigate the independence of pre- and post-metamorphic life-stages and the presence of carry-over effects from one stage to the other, affecting the overall fitness of individuals [5]. The most evident trade-off between the aquatic and terrestrial stages is reflected in body size at metamorphosis. When the aquatic larvae are confronted with unfavourable conditions, such as food shortage [6, 7], high density [8], desiccation risk [9] or predation [10, 11], they can leave the aquatic environment by undergoing metamorphosis. However, this is usually done at a smaller size, which allows them to escape the immediate aquatic threats faster, but in turn exposes them to different selective pressures on land [1].

Larger size in freshly metamorphosed individuals is correlated with improved traits, like locomotor abilities and metabolic rates [12], endurance [13], resistance to desiccation [14], feeding success [15], and dispersal success [16]. Although the paradigm model [17] assumes that body size at metamorphosis is a good predictor of subsequent fitness, there are authors [e.g. 3, 18] who assert that this is not always the case. Since in amphibians a substantial percent of the adult body size is gained after metamorphosis, and because age of sexual maturity is variable, the relationship between body size at metamorphosis and fitness is more complex, especially in unpredictable environments [19]. For example, if smaller individuals have compensating mechanisms, such as more intense growth rates, metamorphic size may not have a significant effect on adult traits like mortality, age and size at first reproduction, or fecundity [20, 21]. The detrimental consequences of a small size at metamorphosis can also be compensated by changes in the morphology of juveniles [22, 23]. For example, the small froglets can have, proportionally to their body size, larger heads or longer legs than the bigger individuals. Such modifications would be beneficial because leg length influences locomotor (i.e. jumping) performance [23, 24], which in turn has been shown to positively affect food acquisition [25], predator avoidance [26] and dispersal [27]. In a similar manner, a large head width favours the swallowing ability, which is a limiting factor in prey selection [28, 29].

In this context, we were interested to understand the influence of size at metamorphosis on post-metamorphic traits such as survival, morphology and performance, and to investigate the existence of compensating mechanisms. Hypothesizing that the difference would be most evident between individuals at the opposite ends of the size range, we evaluated the impact of extreme size phenotypes. We selected as a model a species capable of high plasticity in metamorphosing size [30], the Pacific horned frog (*Ceratophrys stolzmanni*). This fossorial frog inhabits tropical dry forests, a highly seasonal ecosystem characterized by a short rainy season that lasts less than 4 months annually. The tadpoles have some of the most intense growth rates reported for anurans and can leave the water in as little as 2 weeks after egg-laying [30]. Because several environmental parameters can have interacting effects in the natural habitat [31], we used freshly metamorphosed froglets resulting from tadpoles that grew in field conditions (natural ponds) in order to cover the entire range of size at metamorphosis in the population. From the encountered spectrum of sizes, we assigned the 20 largest and 20 smallest individuals to their respective size-categories (see “Methods” section). We tested if smaller froglets can amend the detrimental survival effects induced by metamorphosing at a reduced size through (i) allometric changes in morphology or performance (wider heads, longer limbs, or better jumping skills) and/or (ii) more intense growth rates.

## Results

### Morphology and locomotor performance

At the completion of metamorphosis, individual body mass (BM) and snout-vent length (SVL) showed a strong correlation (Pearson’s *r* = 0.923, *n* = 40, *P* < 0.001), but there was no significant difference in body condition between the two categories of froglets (i.e. small vs. large: *t*_38_ = 0.175, *P* = 0.862; Additional file 1).

**Fig. 1:**
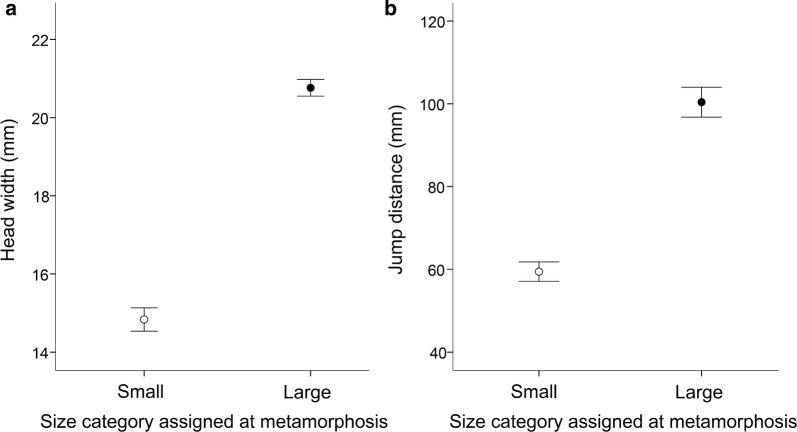
Differences in performance between froglets of the Pacific horned frogs *Ceratophrys stolzmanni* which metamorphosed at small versus large body size (developmental stage Gosner 46; mean ± SE): **a** head width (gape size) and **b** jump distance

Since absolute head width was strongly correlated to SVL at metamorphosis (*r* = 0.982, *n* = 40, *P* < 0.001), larger froglets had significantly wider heads compared to the smaller ones (*t*_38_ = 17.123, *P* < 0.001; Fig. 1a). Relative head width did not differ significantly between the two size categories of froglets (*t*_38_ = − 0.902, *P* = 0.373), and was correlated with individual initial BCI (*r* = 0.479, *n* = 40, *P* = 0.002), with froglets in better body condition having larger heads regardless of their SVL (Additional file 1).

There was a strong positive correlation between hindlimb length and SVL (*r* = 0.988, *n* = 40, *P* < 0.001). Compared to the large juveniles, small froglets had significantly shorter hindlimbs both in terms of absolute size (*t*_38_ = − 17.001, *P* < 0.001), and relative size (*t*_38_ = − 6.309, *P* < 0.001). We found no relationship between the body condition of juveniles and their relative hindlimb lengths (*r* = 0.114, *n *= 40, *P *= 0.485).

Absolute jumping ability was predicted by SVL (*F*_1,36_ = 102.364, *P* < 0.001), with no significant effect of either relative hindlimb length (*F*_1,36_ = 0.539, *P* = 0.468), or BCI (*F*_1,36_ = 0.056, *P* = 0.815). Larger individuals were able to jump over greater distances (mean ± SE = 100.4 ± 3.6 mm) than the smaller ones (59.4 ± 2.3 mm; *t*_38_ = − 9.501, *P *< 0.001; Fig. 1b).

### Growth

At the end of the study period (i.e. 2 months after metamorphosis), the two categories of froglets did not differ in terms of their body condition indices (BCI; *t*_29_ = − 0.289, *P* = 0.774). Individual BCI after 2 months of terrestrial growth was not correlated with the froglet’s SVL at metamorphosis (*r* = 0.107, *n* = 31, *P* = 0.566), nor with initial BCI (*r* = 0.191, *n* = 31, *P* = 0.302).

**Fig. 2:**
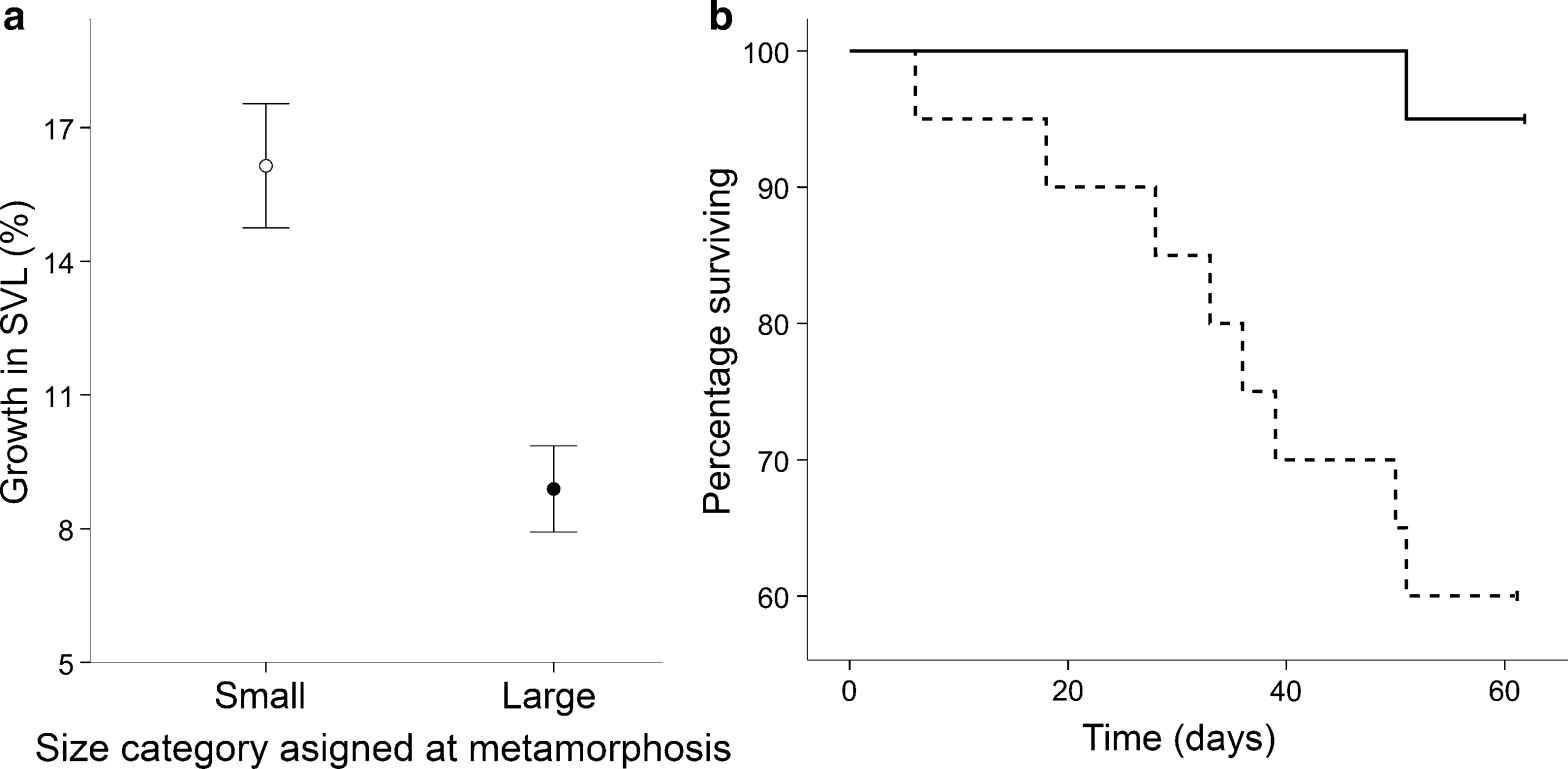
**a** Growth (mean ± SE increase in SVL) of juveniles of Pacific horned frogs *Ceratophrys stolzmanni* that metamorphosed at a small versus a large body size over the study period following metamorphosis (62 days); **b** Kaplan–Meier survival curves for the two groups of juveniles after metamorphosis. Dotted line: small metamorphosing juveniles; full line: large metamorphosing juveniles

There was a significant effect of size at metamorphosis on post-metamorphic growth rates, with individuals that metamorphosed at a smaller size having a higher increase in SVL, both in absolute values (*r*^2^ = 0.31, *F*_1,29_ = 13.054, *P* = 0.001), and in percentage gained by the end of the study (*r*^2^ = 0.566, *F*_1,29_ = 37.782, *P* < 0.001, Fig. 2a). This means that individuals metamorphosing at a small size grew at a significantly higher rate than those metamorphosing at a large size (0.08 mm/day versus 0.06 mm/day on average, respectively; *t*_29_ = 2.29, *P *=0.03). In spite of this, the differences in SVL between the two categories of froglets remained significant until the end of the study (*t*_29_ = − 13.089, *P* < 0.001), although the magnitude decreased (the difference between average SVL in the two groups decreasing from 39% at the start of the study to 27% at the end).

### Mortality

Survivorship over the 2-month study was significantly different between the two categories of froglets: 95% (19 out of 20) of the large individuals survived until the end of the study (i.e. 62 days), compared to 60% (12 out of 20) of the small individuals (log-rank test, *χ*^2^ = 7.318, *P* = 0.007; Fig. 2b). The BCI at metamorphosis was not a predictor of mortality (Cox regression, *χ*^2^ = 0.154, *d.f*. = 1, *P* = 0.695).

## Discussion

Life-history switch-points such as metamorphosis, that require major and irreversible changes in morphology, anatomy, physiology, and habitat and resource use, have a profound effect on individual fitness and involve trade-offs [17, 32, 33]. Our study reveals that individuals making the transition from aquatic larvae to the terrestrial stage at a large size experience higher survival rates during the first activity season. Even under favourable experimental conditions, with no predation or competition, and ad libitum food resources, the juveniles that metamorphosed at a small size had a higher mortality rate. It is expected that in the natural environment, smaller size would result in a further increase in mortality due to exposure to predators [34] and desiccation [14]. Our results imply that, although developmental plasticity can allow tadpoles to escape an unfavourable aquatic environment (e.g. drying pond, high density, reduced food availability) before reaching an optimal size, thus avoiding mortality in the larval stage, it has a direct cost on survival in the terrestrial stage. The lower survival rate of smaller juveniles in terrestrial habitat is consistent with observations made by some authors [6, 15], although in other cases no significant long-term benefits for larger size at metamorphosis in terms of survival was found [21, 35].

Although size at metamorphosis is predicted to have a large impact on size at maturity [32], in some species it was shown that, if environmental conditions are optimal for growth, smaller juveniles can compensate by growing faster and the differences in size eventually fade away [7, 36]. Indeed, our study showed that individuals metamorphosing at a small size are able to increase their growth rate compared to larger individuals, and thus diminish the size gap over time. However, long-term capture-mark-recapture surveys are needed to confirm this pattern in natural conditions.

The existence of compensatory growth (i.e. an increase in growth rates once the conditions are favourable in order to compensate partly or completely the deprivations experienced in younger stages) has been reported in various taxa [37] and can be the result of behavioural modifications, such as prolonged foraging activity and higher ingestion rates [6, 38, 39], or morphological adaptations that allow digestion of larger quantities of food [40]. However, a higher than optimal growth rate comes at a fitness cost, reflected in decreased survival [37]. Along with ecological factors, such as increased exposure to predators and competitors because of prolonged foraging [38], several other components can contribute to the lower fitness associated with accelerated growth rates, amongst which a lower resistance to starvation because of intense metabolism and low lipid reserves [40], delayed ossification [41], depressed immunological function [42], or cellular oxidative stress [43]. Some of the intrinsic costs mentioned above might have contributed to the observed higher mortality in the froglets that had metamorphosed at a small size in our study.

Pacific horned frogs showed one of the widest range of sizes at metamorphosis reported for any amphibian in their natural population, individuals differing by up to 100% in body size and 890% in body mass. This range is broader than previously reported intrapopulation variation in the literature [31, 43]. Pond permanence [30], food availability [44], temperature [45] and presence of predators or competitors [46, 47] are known to have a profound effect on individual metamorphosing size in anurans, and the interaction between various selective pressures can determine a large spectrum of sizes. Additionally, in the case of anuran species reproducing in ephemeral ponds, individuals are less capable to delay their metamorphosis or further increase their larval growth rates, which are already close to the physiological limit [48]. This implies that differences in larval environment will produce a large variation in metamorphic body sizes, such as the one we report here. In the natural habitat, a high diversity of sizes is likely to reduce food competition amongst froglets and allow for a more rapid growth for both large and small individuals [49].

Differences in shape at metamorphosis (i.e. size of different structures in relation to total individual size) are attributed to environmental conditions that promote variations in larval growth rates [23, 50]. Studies carried out on various species give contrasting results, especially in the case of the relationship between hind-limb size (or segments of it) and the individual size [22]. For example, temperature-induced intensification of larval growth rates can generate individuals with relatively shorter legs [22, 24] or longer legs [51]; desiccation-triggered acceleration of growth can determine relatively shorter legs [23], while presence of predators and lack of food can determine slower growth correlated with relatively shorter legs [11]. In our case, metamorphosing at a smaller size was associated with shorter hindlimbs, not only in absolute, but even in relative terms, indicating a departure from isometric size of limbs in this species. Such shorter legs have also been shown to be a result of accelerated development in other species [11, 52].

However, we found that the differences in relative size of hindlimbs did not affect jumping ability in the case of horned frogs; the main factor determining locomotor performance was the body size of juveniles. In fossorial frogs, the leg length in relation to the body size is probably less flexible compared to non-fossorial species of frogs [24, 53], as a result of managing the contrasting selective pressures of avoiding predation or desiccation and efficient burrowing [54]. Alternatively, the variation of leg length might have been too small to determine a measurable difference in jumping distance [55]. We found locomotor performance to be strictly size-dependent, larger individuals having longer legs and jumping over larger distances in response to a simulated predator attack. In anurans, jumping ability is directly related to individual fitness, allowing predator avoidance [26, 34], prey acquisition [25] and dispersal [27].

For gape-limited predators such as amphibians, the width of the head is another morphological trait that can improve fitness, by permitting individuals with wider gape access to larger food items [56]. Since tadpoles of Ceratophryidae are macrophagous, and because their mouth parts go through relatively little restructuration at metamorphosis [57], it can be assumed that the larger relative head width can be an explanation for the improved individual body condition at metamorphosis, regardless of the body size. Overall, head width after metamorphosis was proportional to individual SVL. In the case of strong intra- and inter-specific competition or food shortage, the larger individuals may thus be better equipped for prey ingestion compared to smaller individuals by having access to a wider range of prey sizes. Additionally, since *Ceratophrys* species are known to prey on other amphibians [58], small individuals would also be more at risk of being victims of cannibalistic events. In the cane toad (*Rhinella marina*), the victims of cannibalistic events were a non-random subset of the juvenile population, represented by the smallest and weakest individuals [59]. The lack of beneficial modifications in the proportions of investigated anatomical features, together with the effect of size are likely to act synergistically in nature, decreasing the chances of survival of small metamorphosing individuals, especially in areas with a high density of individuals that increase the probability of cannibalistic encounters.

## Conclusions

Our study links two stages (i.e. aquatic and terrestrial) in amphibian life-history, helping to understand how size at metamorphosis, which is determined by conditions in the aquatic environment, affects the subsequent terrestrial stage, impacting the success and survival of individuals and potentially influencing population dynamics. When aquatic conditions deteriorate, faster metamorphosis is the best survival option for tadpoles, allowing them to take advantage of the terrestrial habitat, where they are able to compensate for smaller size at metamorphosis with an increased growth rate. However, there is a trade-off, as the benefits of leaving water early in life are offset by a lower survival.

## Methods

*Study site and sampling.* On the 16–17 April 2015, we collected 92 freshly metamorphosed *C. stolzmanni*, all in developmental stage Gosner 45 [mouth angle at level of posterior margin of the eye, tail reduced to a stub, 60] in Arenillas Ecological Reserve (03° 34′ S; 80° 08′ E, 30 m a.s.l.), southern Ecuador. This stage was chosen because it allows to select individuals that just exited water at metamorphosis. Because mating is generally synchronized to one night in the studied population [61], we considered that all froglets had approximately the same age. The froglets (*n *= 92) were found in terrestrial habitats, in an area within a 50-m radius from ponds used for reproduction. The average snout-vent length (SVL ± SD) of froglets was 34.5 ± 4.9 mm (range: 23.8–47.9 mm), and average body mass (BM ± SD) was 4.1 ± 1.9 g (range: 1.2–11.9 g). From this spectrum of sizes (Additional file 2), we selected the extreme phenotypes, i.e. the smallest 20 individuals (SVL = 28.21 ± 1.9 mm, range 23.8–30.3 mm; BM = 2.1 ± 0.6 g, range 1.2–3.6) and the largest 20 individuals (SVL = 41.1 ± 2.7 mm, range 38.3–47.9 mm; BM = 6.7 ± 1.6 g, range 5.1–11.9 g).

### Animal care

We raised the froglets in an outdoor laboratory for a period of 62 days (until 24 June 2015), mimicking the duration of an activity season in the natural environment, until the aestivation period (during the dry season), which begins approximately at the end of June (pers. obs.). The froglets were kept individually, in mesh-covered (to prevent escape) plastic tanks (21 × 15 cm, 12 cm high), with a 5-cm layer of moist soil that allowed natural burrowing behaviour [54]. Temperature, relative humidity and light regime were similar to the ones in natural habitat, except for a roof that provided protection from direct sun heating. All tanks were kept in similar conditions, and their relative position was randomly changed after each feeding. The animals were fed an ad libitum, appropriately sized, diet of crickets (similar mixed sizes items for both frog size groups at any time, overall size of crickets slightly larger as the froglets grew). Uneaten insects were removed and fresh food was added every day for the first month, and afterwards every other day until the end of the study. The tanks were checked daily for potential occurrence of death of individuals and sprayed with water to maintain soil humidity. After the completion of the study, on 25 June 2015, all froglets were released at the capture site.

### Morphological traits

The morphometric parameters used in the analyses were SVL (snout-vent length, from the tip of the snout to the cloaca), BM (body mass), head width (at the corners of the mouth), hindlimb length [total hindlimb length, measured as the sum of the right femur, tibia, metatarsus, and the length between the end of the metatarsus to the end of digit IV, 62]. Total hindlimb length in amphibians and its size relative to the total size of the animal is a morphometric trait influencing locomotor ability [63]. Measurements were taken at the beginning (on the 23 April 2015, in G46 developmental stage, i.e. after the complete resorption of the tail so that it does not interfere with movement) and at the end of the study (62 days after the start of the experiment - SVL and BM only), using a Dial-Max calliper (0.1 mm precision) and a My Weigh 300Z portable scale (0.1 g precision). Daily growth rates were calculated as (final SVL − initial SVL)/62. Individuals that died during the study (*n* = 9) were excluded from the growth rate analysis.

### Locomotor performance

Jumping performance tests were taken on 23 April 2015, when individuals had reached developmental stage G46. During the 2 days prior to these trials, the juveniles did not receive any food, so that the presence of food in the digestive system would not influence the results [53]. The lack of feeding during metamorphosis is normal in anurans, due to changes in their digestive system [64]. Jumping performance trials were carried out at a mean ± SE of 22 ± 1 °C, at night-time (21:00–24:00, local time, i.e. during the normal activity hours for the species). Each tested individual (*n* = 40) was placed in the centre of a plastic arena (75 × 45 cm, 33 cm high), lined with a 5 cm layer of moist soil, to provide natural adherence and favour movement [36]; the soil layer being replaced between each trial. Movement (i.e. jumping) was elicited by a gentle touching of the urostyle with a long probe (50 cm long, 1 mm diameter metal stick, with rounded tip), at three times, with a 1-min break between two successive trials. The juvenile was replaced in the centre of the arena before each trial. In the rare occasions when touching elicited a series of multiple movements, only the first jump was measured. The final jumping distance for each individual was the maximum value of the three trials.

### Statistical analysis

Data analysis was carried out with SPSS 21.0 (IBM Corp., Armonk, NY), with a significance level set at 0.05 (dataset available in Additional file 3). Values are given as mean ± SE. All data were first assessed for normality (QQ-plot inspection) and homogeneity of variance (Levene test, *P* > 0.05); some variables (SVL, BM, head width and hindlimb length) were square-transformed to fit the normality assumption. The correlations between various morphological variables were assessed with Pearson’s *r* correlation tests. Linear regression (*F* test) was used to test the effect of size at metamorphosis on growth rates (absolute, percentual) of the froglets. Body condition indexes (BCI) were computed as residuals of lnBM against lnSVL [65]; BCI is considered a good estimate of lipid storage in amphibians [66]. We calculated a relative hindlimb length as the ratio between hindlimb length and SVL, as this was shown to be related to jumping ability [67], and the relative head width as the ratio between head width and SVL. Comparisons of various relevant traits (BCI, head width, hindlimb length, growth rates) between the two size-categories of froglets were investigated using Student’s *t*-tests. To assess if size-free changes in leg size can influence jumping ability, general linear models were built using SVL and size independent traits (relative hindlimb length and BCI) as explanatory variables, and jumping ability as the dependent variable.

Survival rates were calculated as the percentage of individuals that survived until the end of the study in each size-group. The log-rank (Mantel-Cox) test based on Kaplan–Meier survival curves was used to detect differences in survivorship among the two groups, while the influence of body condition on survival probability was tested through a Cox proportional-hazards regression model.

## Supplementary information

**Additional file 1.** Morphometry, locomotor performance traits and growth rates for individuals from the two “metamorphosis” size groups (SVL: snout-vent length). Initial measurement of morphometric traits, as well as jumping trials, were performed in Gosner 46 developmental stage, while final ones were taken 62 days after metamorphosis.

**Additional file 2.** Difference in size at metamorphosis in Pacific horned frogs *Ceratophrys stolzmanni* (developmental stage Gosner 45), *n* = 92. The 20 smallest (white circles) and 20 largest individuals (black dots) were selected to be used in the experiment. The small insert shows a large and a small froglet (photos Diana Székely).

**Additional file 3.** Raw data.

## Data Availability

All data generated or analysed during this study are included in this published article and its supplementary information files (Additional files 1, 2 and 3).

## Abbreviations

SVL: Snout-vent length
BM: Body mass
BCI: Body condition index
