# Supplementary material for: How to recover from a bad start: size at metamorphosis affects growth and survival in a tropical amphibian
Source: BMC Ecol. 2020 Apr 21;20:24. doi: 10.1186/s12898-020-00291-w (PMC7175581; doi:10.1186/s12898-020-00291-w)
Supplement: Supplementary file 1 — Additional file 1. Morphometry, locomotor performance traits and growth rates for individuals from the two “metamorphosis” size groups (SVL: snout-vent length). Initial measurement of morphometric traits, as well as jumping trials, were performed in Gosner 46 developmental stage, while final ones were taken 62 days after metamorphosis. [file 12898_2020_291_MOESM1_ESM.docx]

**Table S1.** Morphometry, locomotor performance parameters and growth rates for individuals from the two “metamorphosis” size groups (SVL: snout-vent length). Initial measurement of morphometric parameters, as well as jumping trials, were performed in Gosner 46 developmental stage, while final ones were taken 62 days after metamorphosis.

| **Parameter** | **Small size at metamorphosis** | | **Large size at metamorphosis** | |
| --- | --- | --- | --- | --- |
|  | **mean ± SE** | **range** | **mean ± SE** | **range** |
| Initial SVL (mm) | 28.7 ± 0.5 (*n* = 20) | 23.8–30.9 | 39.8 ± 0.5 (*n* = 20) | 36.8–45.6 |
| Initial body mass (g) | 2.1 ± 0.1 (*n* = 20) | 1.1–2.9 | 5.8 ± 0.3 (*n* = 20) | 3.9–9.2 |
| Initial Body Condition Index | 0.0024 ± 0.02 (*n* = 20) | -0.151–0.22 | -0.0024 ± 0.02 (*n* = 20) | -0.128–0.117 |
| Head width (mm) | 14.8 ± 0.3 (*n* = 20) | 11.3–16.9 | 20.8 ± 0.2 (*n* = 20) | 19.1–22.8 |
| Relative head width | 0.516 ± 0.006 (*n* = 20) | 0.45–0.58 | 0.522 ± 0.003 (*n* = 20) | 0.496–0.550 |
| Hindlimb length (mm) | 34.7 ± 0.6 (*n* = 20) | 29.3–38.2 | 50.7 ± 0.7 (*n* = 20) | 44.9–56.1 |
| Relative hindlimb length | 1.206 ± 0.008 (*n* = 20) | 1.130–1.266 | 1.274 ± 0.007 (*n* = 20) | 1.217–1.327 |
| Jump distance (mm) | 59.5 ± 2.3 (*n* = 20) | 41–79 | 100.4 ± 3.6 (*n* = 20) | 76–143 |
| Final SVL (mm) | 34.3 ± 0.4 (*n* = 12) | 32.6–36.5 | 43.4 ± 0.4 (*n* = 19) | 40.3–47.4 |
| Final body mass (g) | 4.8 ± 0.3 (*n* = 12) | 2.9–6.3 | 10.5 ± 0.4 (*n* = 19) | 7.7–13.4 |
| Final Body Condition Index | -0.0093 ± 0.04 (*n* = 12) | -0.335–0.134 | 0.0059 ± 0.03 (*n* = 19) | -0.356–0.272 |
| SVL growth (mm) | 4.75 ± 0.39 (*n* = 12) | 3.3–7.35 | 3.49 ± 0.36 (*n* = 19) | 0–5.9 |
| Percentage SVL growth | 16.1 ± 1.4 (*n* = 12) | 11.3–25.2 | 8.9 ± 1 (*n* = 19) | 0–16 |
| Body mass growth (g) | 2.6 ± 0.2 (*n* = 12) | 0.5–3.5 | 4.7 ± 0.5 (*n* = 19) | 1.1–9.2 |
| Percentage body mass growth | 117.8 ± 10.5 (*n* = 12) | 20.8–175 | 88.8 ± 11.1 (*n* = 19) | 12.8–219 |
