# Supplementary figures and images for: How to recover from a bad start: size at metamorphosis affects growth and survival in a tropical amphibian
Source: BMC Ecol. 2020 Apr 21;20:24. doi: 10.1186/s12898-020-00291-w (PMC7175581; doi:10.1186/s12898-020-00291-w)

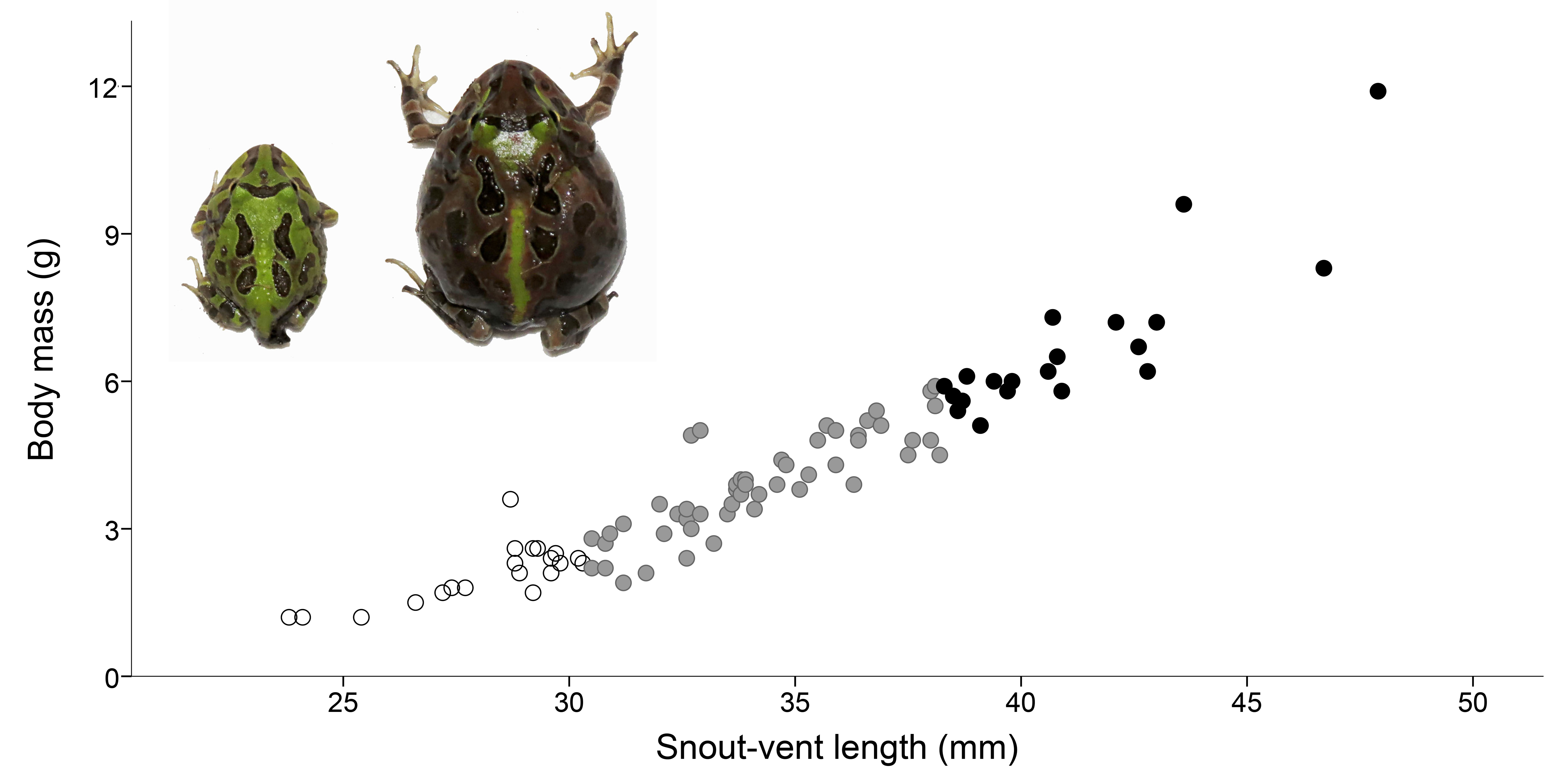

Supplement: Supplementary file 2 — Additional file 2. Difference in size at metamorphosis in Pacific horned frogs Ceratophrys stolzmanni (developmental stage Gosner 45), n = 92. The 20 smallest (white circles) and 20 largest individuals (black dots) were selected to be used in the experiment. The small insert shows a large and a small froglet (photos Diana Székely). [file 12898_2020_291_MOESM2_ESM.jpg]
